# Trajectories of the healthy ageing phenotype among middle-aged and older Britons, 2004–2013

**DOI:** 10.1016/j.maturitas.2016.03.002

**Published:** 2016-06

**Authors:** Gindo Tampubolon

**Affiliations:** Cathie Marsh Institute for Social Research, University of Manchester, Room 2.3E HBS Building, Oxford Road, Manchester M13 9PL, UK

**Keywords:** Biomarkers, Healthy ageing phenotype, Comorbidities, Social determinants, Health behaviours

## Abstract

**Objectives:**

Since the ageing population demands a response to ensure older people remain healthy and active, we studied the dynamics of a recently proposed healthy ageing phenotype. We drew the phenotype’s trajectories and tested whether their levels and rates of change are influenced by health behaviours, comorbidities and socioeconomic positions earlier in the life course.

**Design and outcomes:**

The English Longitudinal Ageing Study, a prospective, nationally representative sample of people aged ≥50 years, measured a set of eight biomarkers which make up the outcome of the healthy ageing phenotype three times over nearly a decade (*N*_2004_ = 5009, *N*_2008_ = 5301, *N*_2013_ = 4455). A cluster of health behaviours, comorbidities and socioeconomic positions were also measured repeatedly. We assessed the phenotype’s distribution non-parametrically, then fitted linear mixed models to phenotypic change and further examined time interactions with gender and socioeconomic position. We ran additional analyses to test robustness.

**Results:**

Women had a wider distribution of the healthy ageing phenotype than men had. The phenotype declined annually by −0.242 (95% confidence interval [CI]: −0.352, −0.131). However, there was considerable heterogeneity in the levels and rates of phenotypic change. Women started at higher levels, then declined more steeply by −0.293 (CI: −0.403, −0.183) annually, leading to crossover in the trajectories. Smoking and physical activity assessed on the Allied Dunbar scale were strongly associated with the trajectories.

**Conclusion:**

Though marked by secular decline, the trajectories of the healthy ageing phenotype showed distinct socioeconomic gradients. The trajectories were also susceptible to variations in health behaviours, strengthening the case for serial interventions to attain healthy and active ageing.

## Highlights

• Women had a wider distribution of the healthy ageing phenotype than men had.
• Women started at higher levels and then declined more steeply, leading to crossover in the trajectories of the healthy ageing phenotype.
• Social gradients in the healthy ageing phenotype were significant.
• Smoking and physical activity were strongly associated with the trajectories.

## Graphical abstract

**Figure:**
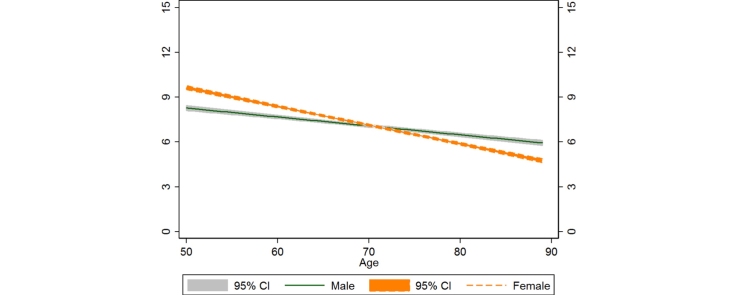

## 1 Introduction

Recently, foundations of healthy ageing based on biomarkers have been proposed on both sides of the Atlantic [1], [2], [3]. Seemingly independent, these proposals nonetheless show considerable overlap and are driven by the same principles of deriving a phenotypic index which captures broad organ systems, while at the same time remaining pragmatically driven by available data in ageing studies. The healthy ageing phenotype, in particular, is sensitive to changes in multiple dimensions that people experience as they age [4], and it captures these by building the phenotype on measures including psychological wellbeing, social wellbeing, physical capability, cognitive function, and physiological and metabolic health [3]. We study one dimension of the phenotype, namely physiological and metabolic health.

Limited work has been done so far on the healthy ageing *phenotype* [3] or the healthy ageing *index* [2]. In the Cardiovascular Health Study, people with higher index scores had significantly lower mortality [1]. With adjustment for demographics, health behaviours and comorbidities, the index significantly predicts death during follow up; and it has been found to be heritable [2].

No empirical study has implemented the healthy ageing phenotype, let alone examined its distribution in the various groups in the population. Such an omission can prove a hindrance to an effective monitoring of and response to the population ageing challenge, for instance to reduce social gradients in health in later life [5]. Thus our first aim is to provide the distribution of the healthy ageing phenotype in the different groups in the population while taking up the suggestion in the proposal to broaden the basis of the phenotype. Next we aim to draw trajectories of phenotypic change from 2004 to 2013 in the British population aged ≥ 50 years using an ongoing, nationally representative ageing study. Most importantly we weigh the contributions of early life course social determinants (including occupation and education) [5], [6], [7], [8], [9] and of contemporaneous comorbidities and health behaviours (smoking, drinking and physical activity) [5], [10] on the levels and rates of change of the healthy ageing phenotype.

We test a number of hypotheses. First, given the well-known social gradients in health, we hypothesise that the phenotype levels will differ along socioeconomic positions throughout the life course, from earlier (education attainment) through more recent (occupation) to contemporaneous (wealth levels) positions. Second, even after accounting for social gradients in the average levels of trajectories in later life there will still be differences in the rates of change according to socioeconomic positions.

## 2 Methods

The English Longitudinal Study of Ageing (ELSA) is the main resource for a nationally-representative ageing study of the English older population. The first wave was in 2002 and subsequent waves follow biennially. Repeated biomarker information is available from the even numbered waves (2004, 2008 and 2012/2013) when nurses visited the participants. The data are freely available from the UK Data Archive (www.data-archive.ac.uk) as study number 5050. More details of the study are given elsewhere [11], [12], [13].

### 2.1 Dependent variable: healthy ageing phenotype

The healthy ageing phenotype was constructed following its definition [3] with an extension to take up a suggestion to broaden its base. These biomarkers as originally proposed include: (*a*) arterial blood pressure as a measure of cardiovascular function, (*b*) fasting glucose and glycated haemoglobin (HbA1C) as markers of glucose homeostasis, (*c*) forced expiratory volume in 1 s as a marker of lung function, (*d*) waist circumference as a marker of adiposity and (*e*) plasma concentrations of HDL cholesterol and triglycerides as markers of lipid metabolism. To this list, another group (*f*) consisting only of serum concentration of high sensitivity C-reactive protein was added. The original definition shared some indicators with the healthy ageing index [2]. The six groups (*a*–*f*), or eight biomarkers, were collected repeatedly and are representative of an array of organ systems.

### 2.2 Collection and assay

Biomarkers data were collected by trained nurses. Systolic blood pressure was measured using a standardised method. Three readings were collected at one-minute intervals using the Omron HEM-907 equipment; the highest reading was used. It was ensured that the room temperature was between 15 °C and 25 °C. Glucose was analysed using the Tosoh Analyzer HLC-723G8. Lung function tests were done using a hand-held Vitalograph spirometer. Three consecutive readings were taken, and the maximum was recorded. Concentrations of HDL cholesterol and triglycerides were measured using Roche Modular Analyzer. High sensitivity C-reactive protein concentration was determined using Roche Modular P. These biomarkers have been described in a number of official ELSA technical reports [14], [15], [16].

We followed the earlier construction [2] by using tertiles or clinical cut-offs (coding 0, 1 and 2 accordingly) of each of the eight biomarkers, then added the codes to give a score which ranges from 0 to 16. The clinical cut-offs for fasting glucose are 6.1 and 7.0 mmol/l (National Institute for Health and Care Excellence; www.nice.org.uk/guidance/ph38/chapter/glossary, accessed 10 August 2015). The cut-offs for waist circumference they are 80 and 88 cm (women) and 94 and 102 cm (men); and for systolic blood pressure they are 130 and 140 mmHg [17]. Being treated for certain diseases coded the related biomarkers to the unhealthiest tertile; as in the instance of receiving treatment for diabetes and its glucose code or receiving treatment for hypertension and its systolic blood pressure code. But we departed from the precedent by reversing the coding to give higher scores for healthier phenotypes.

### 2.3 Covariates

For this first empirical examination of the trajectories, we included an extensive set of covariates. Demographic covariates include gender and age. Since age is capped at 90 in ELSA, information from respondents aged 50–89 was used. Like other health functions, healthy ageing phenotype is also shaped by social determinants of health. These include threefold social class (managerial, intermediate and routine-manual as reference), wealth tertiles (top, middle and bottom as reference), education (some college and high school or less as reference), marital status (married/cohabiting and other as reference).

Based on positive medical history (self-report of ‘has been diagnosed by professionals’), a set of indicators about comorbidities were included, covering cardiovascular diseases of angina, arrythmia, high blood pressure, congestive heart failure, myocardial infarct and heart murmur; chronic obstructive pulmonary disease; diabetes; stroke; arthritis; osteoporosis and cancer. In addition, depression score (Center for Epidemiologic Studies Depression) is included and entered as a continuous variable. Behavioural risk factors known to be important in other studies include smoking (current smoker versus not current smoker as reference), drinking (days in a week having a drink) and physical activity on the Allied Dunbar scale [18] entered as a continuous variable.

Participants with incomplete information on any of the variables were excluded from the analysis, giving an analytic sample of 14,765 observations. Following the “Strengthening the Reporting of Observational Studies in Epidemiology” or STROBE guide [19], a flowchart summarising the included sample is given as a supplement. Differences between those included and excluded were tested using *t*-test (continuous variables) or χ^2^ test (categorical variables). At baseline, compared to those excluded, the analytic sample is younger (65 versus 68 year, p < 0.001), has fewer women (54% versus 57%; p = 0.016), is on average wealthier (£63,310 versus £49,674, p = 0.001) and has healthier ageing phenotype (7.2 versus 5.4, p < 0.001).

### 2.4 Statistical analysis

**Fig. 1:**
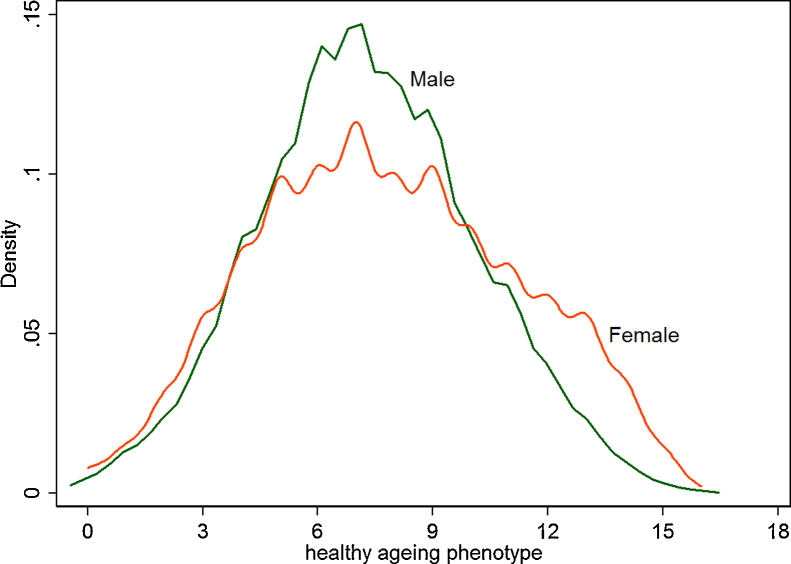
Non-parametric empirical distributions of healthy ageing phenotype for men and women. Source: ELSA 2004–2013.

Since the healthy ageing phenotype distribution was found to be symmetrical and near-normally distributed (see Fig. 1 later), a linear mixed model with random intercepts was fitted to estimate trajectories of change. The random intercepts capture all within individual variations that did not change during study period, including genetic variation. In the baseline model all main effects are included, comprising gender, age, marital status, comorbidities, socioeconomic positions and health behaviours. In the interactions models, in addition to the main effects, interactions between age (in years) with gender, with wealth, with occupation and with education were included separately. Together there were four interactions models. The best model is chosen based on Bayesian Information Criterion [20], picking one with the smallest statistic as the best. All analyses were done in Stata 14 (StataCorp LP, College Station, Texas).

### 2.5 Supplementary analysis: selection model to adjust for dropouts

To deal with attrition bias, arising from participants leaving the study in subsequent waves, an approach based on a selection model is used [21]. In STROBE guide [19], this modelling approach is a form of sensitivity analysis. Here it is implemented in two steps, following the literature [22]. First, we estimated a selection model using information collected in the previous wave to predict dropout. This information includes age, gender, episodic memory, gait speed and health behaviours [23], [24], [9]. From the probability of dropout, an inverse Mills ratio is generated. Next, this ratio and its interactions with time dummies augment a pooled linear model, estimated with panel bootstrap standard errors [22]. We compare the results of linear mixed models with those of attrition-corrected models in a Supplementary table.

## 3 Results

Non-parametric empirical distributions of the healthy ageing phenotype for men and women are shown in Fig. 1. This shows that the distribution for women is wider than that for men; more women than men have higher scores of healthy ageing phenotype while both men and women have similar proportions at lower scores. Importantly, the distributions are symmetric and nearly normal and thus warrant modelling with linear mixed models.

The bivariate relations between healthy ageing phenotype with gender, socioeconomic positions, comorbidities and health behaviours are given in Table 1 which shows that women had higher levels of health ageing phenotype, and that occupation-based and wealth-based gradients are present. Comorbidities distinguish those with higher and lower levels of healthy ageing phenotype such that those with none versus those with three or more comorbidities have more than two-fold difference in levels of healthy ageing phenotype. Health behaviours of smoking and physical activity are able to distinguish those with higher levels of phenotype from those with lower levels, for instance the difference between people with sedentary activities from those with vigorous activity is nearly two points.

**Fig. 2:**
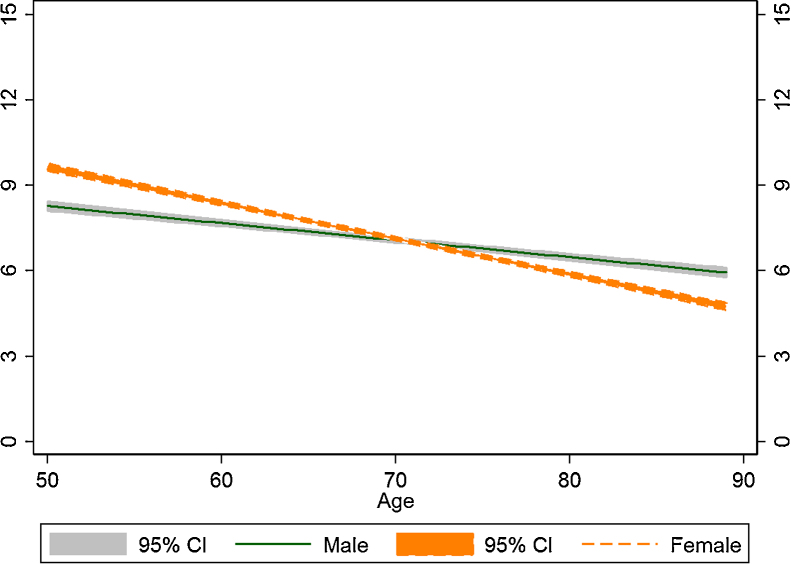
Predicted trajectories and 95% confidence interval (CI) of healthy ageing phenotype for women and men aged ≥ 50 years. Source: ELSA 2004–2013.

The baseline and gender interaction models are compared in Table 2, which shows on the last line that the model with an interaction term between age and gender fits best with the data. In the supplement we show models with interactions terms between socioeconomic positions and gender. Except for this gender interaction term nearly all other interaction terms were not statistically significant. In the best model, namely the gender interaction model, the annual rate of phenotypic decline for men is −0.242 (CI: −0.352, −0.131) but for women this rate is steeper −0.293 (-0.403, −0.183). But the levels of healthy ageing phenotype differ between the gender groups to women’s advantage by 3.691 (2.760, 4.622). Together these patterns of levels and rates make the advantage not to last. Fig. 2 shows the averages of predicted trajectories for men and women, revealing a cross-over in later life, a dynamic revealed here for the first time.

Other notable results from the gender interaction model concern the main effects of socioeconomic positions and behavioural risk factors. Contemporaneous socioeconomic position is captured by wealth. Older people with middle wealth were phenotypically healthier, and the wealthiest were even more so, showing an economic gradient of phenotypic health. Earlier socioeconomic position is captured by occupation which showed an advantage to those in the intermediate occupation relative to those occupying routine manual jobs. An even earlier socioeconomic position is captured by education which showed a net gradient in healthy ageing phenotype with increasing levels of education.

## 4 Discussion

The public health ramifications of population ageing, where there are many more older people enjoying longer lives, are yet to be fully comprehended, and examining a biomarkers-based driver for healthy ageing can help in our understanding. The healthy ageing phenotype, based on a set of biomarkers representing multiple organ systems, is shown for the first time to follow a secular decline from middle age. Also new information is that the experience of decline is sharper for women.

The results on socioeconomic positions reinforced the widely documented social gradients in health outcomes; these also operate at this more basic biomarkers level [5], [25], [7]. Thus the first hypothesis cannot be rejected.

As regards the second hypothesis, the data supported no interaction between the annual rates of decline and any of the socioeconomic positions; the interaction with gender is unique. While women started with a healthier ageing phenotype, their rate of change is steeper such that by the late 80s the advantage has been lost. This dynamic is consistent with the empirical densities shown in Fig. 1, which displayed a more dispersed distribution of women’s phenotypic scores compared to men’s. Gender difference in health function has been suggested before when associations between age on the one hand and allostatic load and metabolic syndrome on the other were examined cross-sectionally [26]. The authors suggest that biological difference between men and women, including differences in hormones, metabolic syndrome and inflammation profiles may be responsible for the difference between men and women in the associations observed. Our findings further suggest that not only the levels (cross-sectionally) but also the rates (longitudinally) were different across gender groups. Further work should explore this longitudinal pattern in different countries with different sets of biomarkers to gain a more complete mechanistic explanation.

While contemporaneous difference in socioeconomic position, indicated by wealth, is evident, it does not fully channel the effects of earlier socioeconomic position. There is still a net association between education attainments much earlier in the life course with healthy ageing phenotype in later life. What matters for older people’s health goes beyond the command of resources afforded by wealth; it includes non-material resources afforded by education. It further raises the need to examine whether education in later life has a potential contribution to healthy ageing phenotype.

Other associations found in the data concern variations that are widely known to be amenable to change in later life: physical activity and smoking. Though entirely unsurprising, it is worth reiterating that the results strongly suggest that broad organ systems are beneficially associated with physical activity and deleteriously related to smoking. In short, the discussion on social determinants and health behaviours suggests that to maintain healthier ageing phenotype in later life, multiple points of intervention, spanning earlier to later life course, should be considered.

This study has various limitations. An important limitation arises from its exclusive use of biomarkers. The healthy ageing phenotype was originally constructed to encompass dimensions beyond physiological and metabolic health [3]. Future work should use a more inclusive conception. Renal function was absent from the healthy ageing phenotype proposal [3]; while in the healthy ageing index from the Cardiovascular Health Study [2] creatinine levels (and Mini-Mental Status Examination scores) were used. Both the phenotype and the index are heavily influenced by available data, and we have been able to expand the healthy ageing phenotype by including a biomarker for inflammation. Nonetheless, by sticking to the proposal this study has been able to examine the dynamics of the phenotype over repeated observations. Beyond the limitation of the phenotype construction, another important limitation concerns the covariates side. Repeated accurate measures of diet are difficult to obtain, even though this is a key contribution to healthy ageing [27]; thus they are missing from this study. A nationally representative sample like ELSA provides a crucial setting for obtaining this measure.

Another limitation arises from the fact that some participants left before the end of the study for various reasons, some of which may relate to their levels of healthy ageing phenotype. This may introduce bias unless the drop out decision is conditionally missing at random, i.e., the decision is related only to those factors accounted for. Maximum likelihood estimator of linear mixed model used here is known to yield consistent estimates under missing at random assumption [20]. Nevertheless, following the STROBE guide [19], an additional sensitivity analysis using a selection correction model of dropout [22] gave encouraging results (Supplementary table). In the dropout-corrected model, the coefficients are variously attenuated while consistently retaining their signs and significance values. This consistency suggests that results are robust: higher levels of healthy ageing phenotype among women to begin with, followed by steeper rates of decline.

The dynamics of healthy ageing phenotype also need to be examined across different countries, as might be discerned from the motivation for this study. The construct of the healthy ageing phenotype has a broader provenance beyond Britain [3] and it was motivated by the complexity of the ageing process.

A seminal work on healthy or successful ageing [28] recognises this complexity, highlighting the importance of health behaviours while also exploring the physio-metabolic basis of healthy ageing. Recent studies on ELSA have shown for example how behaviours, including physical activity and regular drink, are associated with lung and cognitive functions [10], [29]. The results of our study suggest that the phenotype, with a more updated list of biomarkers, is potentially susceptible to health behaviours interventions.

The complexity of the healthy ageing process also motivated the multiple dimensions that make up the original proposal for the phenotype [3]. Beyond suggesting broadening the dimensions that should be measured at one time if one is to gain a better grasp of healthy ageing, recent scholarship has suggested that focusing on multiple times through the life course is likely to be a fruitful approach to understand the rich experience of healthy ageing. Kuh et al. [6] have delineated different phases of the life course beyond those examined in this study, that can contribute to healthy ageing. The life course approach embraces earlier periods including in utero. A recent study of ELSA has shown, for example, that conditions when children turned ten relate to their gait speed, memory function and depression up to eight decades later [9].

This work has a number of strengths. This is the first implementation of this proposal and it has made use of repeated measurement from an ongoing longitudinal study of ageing. Therefore further investigation or refinement is not far-fetched. Also three measures of complete biomarkers are rare, especially those that closely map to a theoretical construct as proposed [3]. Another strength comes from the nationally representative nature of ELSA and its broad spans from middle to later life periods; this ensures the patterns uncovered can be generalised without being unduly limited by the specific characteristics in some volunteer or otherwise selected samples.

In conclusion, although the biomarkers-based indicator of healthy ageing is partly heritable, there are evidently multiple points of intervention throughout life that can alter the course of the physiological and metabolic basis of ageing. This biological basis can still benefit from changes in behaviours, such as engaging in physical activity, which can be enacted by individuals and facilitated by public health investment to ensure active and healthy ageing.

## Contributors

GT was the sole author.

## Conflict of interest

None declared.

## Funding

The funding bodies were not involved in the design, analysis, writing and publication of this study.

## Ethical approval

Ethical approval for all the ELSA waves was granted from the National Research and Ethics Committee of the UK National Health Service (www.nres.nhs.uk). The University’s institutional review board has approved this study in its use of publicly available anonymised secondary data.

## Provenance and peer review

This article has undergone peer review.

## Figures and Tables

**Table 1:** Summary statistics of mean (standard error [SE]) levels of healthy ageing phenotype across gender and different groups. Source: ELSA 2004–2013.

	Male		Female		Total	p Value
	Mean	SE Mean	Mean	SE Mean	Mean	
Age group						0.50
50- (n = 4109)	7.90	(0.08)	8.97	(0.09)	8.51	
60- (n = 5843)	7.45	(0.07)	7.82	(0.07)	7.65	
70- (n = 3648)	6.50	(0.08)	6.51	(0.08)	6.51	
80- (n = 1165)	2.92	(0.35)	2.52	(0.37)	2.69	

Marital status						<0.01
Single (n = 818)	7.56	(0.18)	7.77	(0.22)	7.65	
Married (n = 10,210)	7.35	(0.05)	8.02	(0.06)	7.69	
Separated (n = 1663)	7.32	(0.15)	7.87	(0.13)	7.68	
Widowed (n = 2074)	6.19	(0.19)	6.77	(0.12)	6.65	

Occupation						<0.01
Managerial (n = 5826)	7.58	(0.06)	8.45	(0.08)	7.99	
Intermediate (n = 3505)	7.44	(0.15)	7.79	(0.08)	7.72	
Routine manual (n = 5434)	6.95	(0.07)	7.05	(0.09)	7.00	

Wealth tertiles						0.02
Bottom (n = 4135)	6.58	(0.09)	6.74	(0.09)	6.67	
Middle (n = 5089)	7.19	(0.08)	7.62	(0.08)	7.43	
Wealthiest (n = 5541)	7.80	(0.07)	8.69	(0.08)	8.27	

Education						< 0.01
<High school (n = 4561)	6.45	(0.09)	6.70	(0.08)	6.61	
High school (n = 5692)	7.35	(0.07)	8.00	(0.07)	7.72	
College (n = 4512)	7.76	(0.07)	8.81	(0.10)	8.23	

Current smoker						0.96
No (n = 12,979)	7.40	(0.05)	7.89	(0.05)	7.66	
Yes (n = 1786)	6.53	(0.13)	7.01	(0.15)	6.79	
Total (n = 14,765)	7.30	(0.04)	7.79	(0.05)	7.57	

Drinking						<0.01
Less (n = 9098)	6.90	(0.06)	7.31	(0.06)	7.15	
Daily (n = 5667)	7.72	(0.06)	8.70	(0.08)	8.17	

Physical activity (Allied Dunbar scale)						<0.01
Less (n = 5701)	6.69	(0.08)	7.03	(0.08)	6.90	
Moderate (n = 6325)	7.30	(0.06)	7.97	(0.07)	7.66	
High (n = 2739)	8.12	(0.09)	9.07	(0.12)	8.56	

Angina, arrythmia, hypertensive, congestive heart failure, myocardial infarct, heart murmur						<0.01
No (n = 8602)	8.47	(0.06)	9.33	(0.06)	8.96	
Yes (n = 6163)	5.75	(0.05)	5.36	(0.05)	5.55	

COPD						0.65
No (n = 13,348)	7.48	(0.05)	7.96	(0.05)	7.74	
Yes (n = 1417)	5.58	(0.14)	6.15	(0.14)	5.89	

Diabetes						<0.01
No (n = 14,187)	7.62	(0.04)	8.06	(0.05)	7.86	
Yes (n = 578)	3.44	(0.12)	2.61	(0.13)	3.07	

Cancer						0.05
No (n = 14,193)	7.33	(0.05)	7.81	(0.05)	7.59	
Yes (n = 572)	6.41	(0.22)	7.34	(0.26)	6.94	

Stroke						0.01
No (n = 14,539)	7.34	(0.04)	7.82	(0.05)	7.61	
Yes (n = 226)	5.24	(0.28)	4.98	(0.37)	5.13	

Arthritis, osteoporosis						<0.01
No (n = 11,943)	7.44	(0.05)	8.01	(0.06)	7.74	
Yes (n = 2822)	6.54	(0.12)	7.04	(0.10)	6.86	

Cancer						0.05
No (n = 14,193)	7.33	(0.05)	7.81	(0.05)	7.59	
Yes (n = 572)	6.41	(0.22)	7.34	(0.26)	6.94	

CES-D						<0.01
Not depressed (n = 13,023)	7.37	(0.05)	7.93	(0.05)	7.67	
Depressed (n = 1742)	6.44	(0.17)	6.88	(0.13)	6.74	
Total (n = 14,765)	7.30	(0.04)	7.79	(0.05)	7.57	

**Table 2:** Baseline and interaction models between age with gender, coefficients and 95% confidence intervals. Source: ELSA 2004–2013.

	Baseline	With gender
	β [CI]	β [CI]
Sex, female	0.416^***^	3.691^***^
	[0.287,0.545]	[2.760,4.622]
Age	−0.276^***^	−0.242^***^
	[−0.387,−0.166]	[−0.352,−0.131]
Non-smoker (reference)		
Smoker	−0.679^***^	−0.685^***^
	[−0.852,−0.507]	[−0.858,−0.513]
Less than daily (reference)		
Drink daily	0.414^***^	0.412^***^
	[0.309,0.519]	[0.307,0.517]
Physical activity	0.240^***^	0.237^***^
	[0.176,0.303]	[0.174,0.301]
Occupation		
Routine manual (reference)		
Intermediate	0.114	0.137
	[−0.052,0.280]	[−0.028,0.303]
Managerial	0.072	0.070
	[−0.087,0.231]	[−0.089,0.228]
Education		
<High school (reference)		
High school	0.367^***^	0.342^***^
	[0.216,0.519]	[0.190,0.493]
College	0.588^***^	0.582^***^
	[0.405,0.771]	[0.399,0.765]
Wealth tertiles		
Bottom third (reference)		
Middle wealth	0.303^***^	0.298^***^
	[0.174,0.432]	[0.169,0.426]
Wealthiest	0.645^***^	0.641^***^
	[0.506,0.785]	[0.502,0.780]
Age × Female		−0.051^***^
		[−0.065,−0.037]
*N*	14765	14765
Adj. *R*^2^	0.44	0.45
BIC	36871	36828

Models were adjusted for marital status, comorbidities including cardiovascular diseases (angina, arrythmia, high blood pressure, congestive heart failure, myocardial infarct and heart murmur); chronic obstructive pulmonary disease; diabetes; stroke; arthritis; osteoporosis; cancer; depression. CI: 95% confidence intervals. ^*^p < 0.05, ^**^p < 0.01, ^***^p < 0.001.
