# Supplementary material for: Trajectories of the healthy ageing phenotype among middle-aged and older Britons, 2004–2013
Source: Maturitas. 2016 Jun;88:9–15. doi: 10.1016/j.maturitas.2016.03.002 (PMC4850932; doi:10.1016/j.maturitas.2016.03.002)
Supplement: Supplementary file 1 [file mmc1.docx]

Table S1. Baseline characteristics, 2004

|  | Male |  | Female |  |
| --- | --- | --- | --- | --- |
|  | Mean | SE | Mean | SE |
| Healthy ageing phenothype | 6.95 | 0.08 | 7.48 | 0.09 |
| Blood pressure, *mmHg* | 135.71 | 0.39 | 134.00 | 0.39 |
| Fasting glucose, *mmol/l* | 5.13 | 0.03 | 4.92 | 0.02 |
| Glycated haemoglobin, *mmol/mol* | 35.79 | 0.35 | 35.71 | 0.27 |
| Forced expiratory volume 1 sec, *l* | 2.81 | 0.02 | 2.00 | 0.01 |
| Waist circumference, *cm* | 101.03 | 0.24 | 90.10 | 0.23 |
| High density lipoprotein, *mmol/l* | 1.38 | 0.1 | 1.66 | 0.01 |
| Triglyceride, *mmol/l* | 1.94 | 0.03 | 1.71 | 0.02 |
| C-reactive protein, *mg/l* | 2.50 | 0.05 | 2.74 | 0.05 |
|  |  |  |  |  |
| Age | 65.28 | 0.19 | 65.56 | 0.18 |
| Marital status |  |  |  |  |
| Single (n=248) | 7.52 | 0.32 | 7.32 | 0.43 |
| Married (n=3,469) | 7.01 | 0.08 | 7.71 | 0.10 |
| Sepdiv (n=510) | 7.06 | 0.26 | 7.56 | 0.25 |
| Widowed (n=782) | 5.58 | 0.33 | 6.56 | 0.20 |
| Occupation |  |  |  |  |
| Managerial (n=1,817) | 7.21 | 0.11 | 8.03 | 0.16 |
| Intermediate (n=1,220) | 7.08 | 0.25 | 7.61 | 0.14 |
| Routine else (n=1,972) | 6.68 | 0.11 | 6.83 | 0.14 |
| Wealth tertiles |  |  |  |  |
| Bottom (n=1,405) | 6.20 | 0.16 | 6.29 | 0.15 |
| Middle (n=1,720) | 6.88 | 0.13 | 7.49 | 0.14 |
| Wealthiest (n=1,884) | 7.46 | 0.11 | 8.37 | 0.14 |
| Education |  |  |  |  |
| < High school (n=1,802) | 6.27 | 0.14 | 6.57 | 0.14 |
| High school (n=1,916) | 7.01 | 0.12 | 7.86 | 0.13 |
| College (n=1,291) | 7.46 | 0.13 | 8.40 | 0.20 |
| Current smoker |  |  |  |  |
| No (n=4,335) | 7.05 | 0.08 | 7.56 | 0.09 |
| Yes (n=674) | 6.35 | 0.20 | 6.84 | 0.26 |
| Drinking |  |  |  |  |
| Less (n=3,090) | 6.38 | 0.11 | 6.99 | 0.10 |
| Daily (n=1,919) | 7.59 | 0.10 | 8.43 | 0.14 |
| Physical activity (Allied Dunbar) |  |  |  |  |
| Less (n=1,238) | 5.36 | 0.18 | 5.90 | 0.16 |
| Moderate (n=2,701) | 7.04 | 0.10 | 7.75 | 0.11 |
| High (n=1,070) | 7.94 | 0.14 | 8.70 | 0.19 |
| Angina, arrythmia, high BP, CHF, myocardial infarct, heart murmur |  |  |  |  |
| No (n=2,544) | 8.37 | 0.10 | 9.49 | 0.11 |
| Yes (n=2,465) | 5.60 | 0.09 | 5.44 | 0.09 |
| COPD |  |  |  |  |
| No (n=4,165) | 7.23 | 0.08 | 7.74 | 0.10 |
| Yes (n=844) | 5.48 | 0.18 | 6.20 | 0.18 |
| Diabetes |  |  |  |  |
| No (n=4,628) | 7.56 | 0.07 | 7.99 | 0.08 |
| Yes (n=381) | 3.24 | 0.13 | 2.38 | 0.16 |
| Cancer |  |  |  |  |
| No (n=4,660) | 7.02 | 0.08 | 7.48 | 0.09 |
| Yes (n=349) | 5.77 | 0.29 | 7.40 | 0.34 |
| Stroke |  |  |  |  |
| No (n=4,845) | 7.03 | 0.08 | 7.55 | 0.09 |
| Yes (n=164) | 5.11 | 0.34 | 4.71 | 0.45 |
| Arthritis, osteoporosis |  |  |  |  |
| No (n=3,049) | 7.14 | 0.09 | 8.07 | 0.11 |
| Yes (n=1,960) | 6.48 | 0.15 | 6.76 | 0.13 |
| Cancer |  |  |  |  |
| No (n=4,660) | 7.02 | 0.08 | 7.48 | 0.09 |
| Yes (n=349) | 5.77 | 0.29 | 7.40 | 0.34 |
| CES-D |  |  |  |  |
| Not depressed (n=4,362) | 7.04 | 0.08 | 7.64 | 0.09 |
| Depressed (n=647) | 6.01 | 0.28 | 6.53 | 0.21 |
