# Supplementary material for: Trajectories of the healthy ageing phenotype among middle-aged and older Britons, 2004–2013
Source: Maturitas. 2016 Jun;88:9–15. doi: 10.1016/j.maturitas.2016.03.002 (PMC4850932; doi:10.1016/j.maturitas.2016.03.002)
Supplement: Supplementary file 2 [file mmc2.docx]

Table S2. Interaction models between age with socioeconomic positions, coefficients and 95% confidence intervals. Source: ELSA 2004-2013.

|  | With occupation | With education | With wealth |
| --- | --- | --- | --- |
|  | β [CI] | β [CI] | β [CI] |
| Sex, Female | 0.423^***^ | 0.417^***^ | 0.415^***^ |
|  | [0.293,0.552] | [0.288,0.547] | [0.286,0.544] |
|  |  |  |  |
| Age | -0.292^***^ | -0.292^***^ | -0.273^***^ |
|  | [-0.403,-0.180] | [-0.406,-0.177] | [-0.383,-0.162] |
| Occupation |  |  |  |
| Routine manual (reference) |  |  |  |
| Intermediate | 0.388 | 0.117 | 0.114 |
|  | [-0.827,1.604] | [-0.050,0.283] | [-0.052,0.280] |
|  |  |  |  |
| Managerial | -1.319^*^ | 0.074 | 0.073 |
|  | [-2.386,-0.251] | [-0.085,0.233] | [-0.086,0.231] |
| Education |  |  |  |
| < high school (reference) |  |  |  |
| High school | 0.357^***^ | -0.054 | 0.370^***^ |
|  | [0.205,0.509] | [-1.220,1.112] | [0.218,0.521] |
|  |  |  |  |
| College | 0.588^***^ | -0.079 | 0.588^***^ |
|  | [0.405,0.772] | [-1.327,1.170] | [0.405,0.771] |
| Wealth tertiles |  |  |  |
| Bottom third (reference) |  |  |  |
| Middle wealth | 0.306^***^ | 0.303^***^ | 0.011 |
|  | [0.177,0.435] | [0.174,0.432] | [-1.044,1.065] |
|  |  |  |  |
| Wealthiest | 0.645^***^ | 0.646^***^ | 1.206^*^ |
|  | [0.506,0.785] | [0.506,0.785] | [0.135,2.277] |
| ˟ | -0.004 |  |  |
| Intermediate | [-0.023,0.014] |  |  |
|  |  |  |  |
| ˟ Managerial | 0.022^**^ |  |  |
|  | [0.005,0.038] |  |  |
| ˟ High |  | 0.006 |  |
| school |  | [-0.011,0.024] |  |
| ˟ College |  | 0.010 |  |
|  |  | [-0.009,0.029] |  |
| ˟ Middle |  |  | 0.004 |
| wealth |  |  | [-0.012,0.021] |
| ˟ Wealthiest |  |  | -0.009 |
|  |  |  | [-0.025,0.008] |
| *N* | 14765 | 14765 | 14765 |
| Adj. *R*^2^ | 0.44 | 0.44 | 0.44 |
| BIC | 36887 | 36877 | 36884 |

All models were adjusted with marital status, comorbidities including cardiovascular diseases (angina, arrythmia, high blood pressure, congestive heart failure, myocardial infarct and heart murmur); chronic obstructive pulmonary disease; diabetes; stroke; arthritis; osteoporosis; cancer; depression, smoking, drinking and physical activity.

CI: 95% confidence intervals

^*^ *p* < 0.05, ^**^ *p* < 0.01, ^***^ *p* < 0.001
