# Supplementary material for: Trajectories of the healthy ageing phenotype among middle-aged and older Britons, 2004–2013
Source: Maturitas. 2016 Jun;88:9–15. doi: 10.1016/j.maturitas.2016.03.002 (PMC4850932; doi:10.1016/j.maturitas.2016.03.002)
Supplement: Supplementary file 3 [file mmc3.docx]

Table S3. Sensitivity analysis (final and selection models)

|  | Final model | Selection model |
| --- | --- | --- |
|  | β [CI] | β [CI] |
| She, Female | 3.691^***^ | 2.545^***^ |
|  | [2.760,4.622] | [1.446,3.644] |
| Age | -0.242^***^ | -0.028^***^ |
|  | [-0.352,-0.131] | [-0.043,-0.013] |
| Non-smoker (reference) |  |  |
| Current smoker | -0.685^***^ | -0.672^***^ |
|  | [-0.858,-0.513] | [-0.909,-0.435] |
| Less than daily (reference) |  |  |
| Drink daily | 0.412^***^ | 0.509^***^ |
|  | [0.307,0.517] | [0.359,0.658] |
| Physical activity | 0.237^***^ | 0.374^***^ |
|  | [0.174,0.301] | [0.285,0.463] |
| Occupation |  |  |
| Routine manual (reference) |  |  |
| Intermediate | 0.137 | 0.161 |
|  | [-0.028,0.303] | [-0.059,0.381] |
| Managerial | 0.070 | -0.009 |
|  | [-0.089,0.228] | [-0.190,0.172] |
| Education |  |  |
| < high school (reference) |  |  |
| High school | 0.342^***^ | 0.270^**^ |
|  | [0.190,0.493] | [0.086,0.454] |
| College | 0.582^***^ | 0.449^***^ |
|  | [0.399,0.765] | [0.223,0.676] |
| Wealth tertiles |  |  |
| Bottom third (reference) |  |  |
| Middle wealth | 0.298^***^ | 0.339^***^ |
|  | [0.169,0.426] | [0.169,0.508] |
| Wealthiest | 0.641^***^ | 0.705^***^ |
|  | [0.502,0.780] | [0.510,0.900] |
| Age˟ Female | -0.051^***^ | -0.035^***^ |
|  | [-0.065,-0.037] | [-0.051,-0.019] |
| *N* | 14814 | 14814 |
| adj. *R*^2^ | 0.45 | 0.44 |

Both models were adjusted with marital status, comorbidities including cardiovascular diseases (angina, arrythmia, high blood pressure, congestive heart failure, myocardial infarct and heart murmur); chronic obstructive pulmonary disease; diabetes; stroke; arthritis; osteoporosis; cancer; depression.

95% confidence intervals in brackets

^*^ *p* < 0.05, ^**^ *p* < 0.01, ^***^ *p* < 0.001
