# Supplementary material for: Trajectories of the healthy ageing phenotype among middle-aged and older Britons, 2004–2013
Source: Maturitas. 2016 Jun;88:9–15. doi: 10.1016/j.maturitas.2016.03.002 (PMC4850932; doi:10.1016/j.maturitas.2016.03.002)
Supplement: Supplementary file 4 [file mmc4.docx]

Excluded for missing biomarkers (systolic blood pressure, fasting glucose, glycated haemoglobin, forced expiratory volume, waist circumference, high density lipoprotein, triglycerides, C-reactive protein).

- 2004 n = 2487
- 2008 n = 3017
- 2012 n = 2645

Excluded for missing socioeconomic and health variables (marital status, comorbidities including cardiovascular diseases, chronic obstructive pulmonary disease, diabetes, stroke, arthritis, osteoporosis, cancer, depression, smoking, drinking and physical activity).

- 2004 n = 67
- 2008 n = 143
- 2012 n = 830

Available

- 2004 n = 7563
- 2008 n = 8461
- 2012 n= 7930

Eligible

- 2004 n = 7496
- 2008 n = 8318
- 2012 n= 7100

Analytic sample

- 2004 n = 5009
- 2008 n = 5301
- 2012 n= 4455

Flowchart of analytic sample preparation
